# Activation of CNTF/CNTFRα Signaling Pathway by hRheb(S16H) Transduction of Dopaminergic Neurons *In Vivo*

**DOI:** 10.1371/journal.pone.0121803

**Published:** 2015-03-23

**Authors:** Kyoung Hoon Jeong, Jin Han Nam, Byung Kwan Jin, Sang Ryong Kim

**Affiliations:** 1 School of Life Sciences, Institute of Life Science & Biotechnology, Kyungpook National University, Daegu, Korea; 2 BK21 plus KNU Creative BioResearch Group, Institute of Life Science & Biotechnology, Kyungpook National University, Daegu, Korea; 3 Institute of Life Science & Biotechnology, Kyungpook National University, Daegu, Korea; 4 Neurodegeneration Control Research Center, School of Medicine, Kyung Hee University, Seoul, Korea; 5 Department of Biochemistry & Molecular Biology, School of Medicine, Kyung Hee University, Seoul, Korea; 6 Brain Science and Engineering Institute, Kyungpook National University, Daegu, Korea; University of Florida, UNITED STATES

## Abstract

Ciliary neurotrophic factor (CNTF) is one of representative neurotrophic factors for the survival of dopaminergic neurons. Its effects are primarily mediated via CNTF receptor α (CNTFRα). It is still unclear whether the levels of CNTFRα change in the substantia nigra of Parkinson’s disease (PD) patients, but CNTF expression shows the remarkable decrease in dopaminergic neurons in the substantia nigra pars compacta (SNpc), suggesting that the support of CNTF/CNTFRα signaling pathway may be a useful neuroprotective strategy for the nigrostriatal dopaminergic projection in the adult brain. Here, we report that transduction of rat SNpc dopaminergic neurons by adeno-associated virus with a gene encoding human ras homolog enriched in brain (hRheb), with an S16H mutation [hRheb(S16H)], significantly upregulated the levels of both CNTF and CNTFRα in dopaminergic neurons. Moreover, the hRheb(S16H)-activated CNTF/CNTFRα signaling pathway was protective against 1-methyl-4-phenylpyridinium-induced neurotoxicity in the nigrostriatal dopaminergic projections. These results suggest that activation of CNTF/CNTFRα signaling pathway by specific gene delivery such as hRheb(S16H) may have therapeutic potential in the treatment of PD.

## Introduction

Ciliary neurotrophic factor (CNTF), a neurotrophic cytokine belonging to the interleukin-6 family, is expressed in diverse brain regions [1]. CNTF regulates several functions, such as gene expression, cell survival, and differentiation in a variety of neuronal cells [2, 3]. These are mediated by tripartite receptor complexes, such as leukemia inhibitory factor receptor β, glycoprotein 130, and CNTF-specific ligand-binding receptor α-subunit (CNTFRα) [4]. Expression of CNTF and CNTFRα can increase in the brain following injury [5–7]; however, the levels of CNTF are reduced in dopaminergic neurons in the substantia nigra (SN) of Parkinson’s disease (PD) patients [8] and treatment with recombinant human CNTF preventes the degeneration of dopaminergic neurons [9]. Moreover, the loss of neurotrophic factors such as glial cell line-derived neurotrophic factor (GDNF) and brain-derived neurotrophic factor (BDNF) with CNTF may be involved in the pathogenesis of brain diseases [8, 10, 11]. These observations suggest that the support of neurotrophic factors, such as CNTF, GDNF, and BDNF, may be useful to protect neurons against neurodegenerative diseases, and their induction can be potential therapeutic targets for PD.

In a previous study, we have shown that transduction of human ras homolog enriched in brain (hRheb) with a mutation of serine to histidine at position 16 [hRheb(S16H)] exerted neurotrophic effects in dopaminergic neurons via the activation of mammalian target of rapamycin complex 1 (mTORC1), resulting in the protection and restoration of the nigrostriatal dopaminergic projection in a neurotoxin model of PD [12, 13]. Furthermore, we have recently reported that hRheb(S16H) expression could induce the production of BDNF and GDNF, which is neuroprotective in dopaminergic neurons in a neurotoxin model of PD [14]. These results suggest that activation of the signaling pathways involved in cell survival by a specific gene delivery, such as hRheb(S16H), may be a useful strategy for protecting the nigrostriatal dopaminergic system in the adult brain.

It is still unclear whether hRheb(S16H) transduction of dopaminergic neurons can induce the production of additional survival factors such as CNTF and CNTFRα, which are also involved in protection of the nigrostriatal dopaminergic projection in the adult brain. Therefore, in the present study, we examined whether hRheb(S16H) as an activator for production of CNTF and CNTFRα and if this contributed to neuroprotection against 1-methyl-4-phenylpyridinium (MPP^+^)-induced neurotoxicity on the nigrostriatal dopaminergic projection in the adult rat brain.

## Materials and Methods

### Institutional review of animal protocols

Forty Sprague Dawley rats (10 weeks old, 220–240 g) were obtained from Daehan Biolink (Eumseong, Korea). Rats were housed in a controlled environment (temperature, 22 ± 1°C; humidity, 50 ± 1%) and fully provided with food and water. All surgical experiments were performed in accordance with approved animal protocols and guidelines established by the Animal Care Committee of Kyungpook National University (No. KNU 2012–37). All surgeries were performed under anesthesia by intraperitoneal injection of chloral hydrate (360 mg/kg; Sigma, St. Louis, MO), and all efforts were made to minimize suffering and reduce the number of animals used.

### Production of adeno-associated virus (AAV) viral vectors

All vectors used in these studies were AAV1 serotype. AAVs with a gene encoding hRheb(S16H) or green fluorescent protein (GFP), used as a control, were produced by the University of North Carolina Vector Core as previously described [12–14]. The genomic titer of hRheb(S16H) and GFP were 2 × 10^12^ and 1 × 10^12^ viral genomes/ml, respectively.

### Intranigral AAV injection

Rats received a unilateral injection of AAV-GFP or AAV-hRheb(S16H) into the right SN as previously described [14]. Coordinates were calculated according to Paxinos and Watson [15] (AP: - 6.0 mm; ML: - 2.3 mm; DV: - 7.6 mm, relative to the bregma). After the injection, the needle was left in place for an additional 5 min before being slowly retracted. The viral vector suspension (2.0 μl) was injected at a rate of 0.1 μl/min over 20 min.

### Intranigral neutralizing antibody injection and intra-medial forebrain bundle (MFB) MPP^+^ injection

CNTF or CNTFRα neutralizing antibodies (200 ng in 2 μl, respectively; R&D systems, Minneapolis, MN) were stereotaxically injected at a rate of 0.5 μl/min into the right SN (AP: - 5.8 mm; ML: - 2.3 mm; DV: - 7.6 mm), and then MPP^+^ [7.4 μg in 2 μl phosphate-buffered saline (PBS); Sigma] was injected at a rate of 0.5 μl/min into the right MFB (AP: - 3.6mm; ML: - 2.0mm; DV: - 7.5mm), as described previously [16]. Rats that were injected with neutralizing antibodies in the absence of hRheb(S16H) were used as controls.

### Immunohistochemical staining procedures

Rat tissues were prepared for immunostaining as previously described [14]. Sections were incubated with anti-CNTF (1:000; R&D systems), anti-CNTFRα (1:000; R&D systems), anti-FLAG (1:2000; Sigma), or anti-tyrosine hydroxylase (TH, 1:2000; Pel-Freez, Rogers, AR) overnight at 4°C and then incubated with biotin-conjugated secondary antibodies, followed by an avidin-biotin complex kit (Vector Laboratories, Burlingame, CA). The signal was detected by incubating sections in 0.5 mg/ml 3,3’-diaminobenzidine (DAB; Sigma) in 0.1 M phosphate buffer (PB; Sigma) containing 0.003% H_2_O_2_. Sections were analyzed under a microscope (Axio Imager; Carl Zeiss, Göttingen, Germany). For immunofluorescence labeling, brain sections were incubated overnight with one of the following pairs: anti-FLAG (1:2000; Sigma) and anti-TH (1:2000; Pel-Freez), anti-TH (1:2000; Pel-Freez) and anti-CNTF (1:000; R&D systems), or anti-TH (1:2000; Pel-Freez) and anti-CNTFRα (1:000; R&D systems). The next day, sections were incubated with Cy3- (1:200; Millipore, Bedford, MA) and FITC-conjugated IgG (1:200; Millipore) for 1 h, and then washed and mounted with Vectashield mounting medium (Vector Laboratories). Sections were analyzed under a bright-field microscope (Axio Imager; Carl Zeiss) or with confocal microscopy (LSM700; Carl Zeiss).

### Stereological estimation

As previously described [16–19], the total number of TH-positive neurons was counted in the various animal groups using the optical fractionator method performed on an bright-field microscope (Olympus Optical, BX51, Tokyo, Japan) using Stereo Investigator software (MBF Bioscience, Williston, VT). Dopaminergic neurons in the SN were quantitatively expressed as a percentage of the number of TH-positive neurons in the ipsilateral side compared to the contralateral control side.

### Quantitative determination of striatal TH immunoperoxidase staining

The densitometric analysis in the rat striatum (STR) was examined as previously described [12, 14, 20]. TH-positive fiber innervation in the STR was quantitatively expressed as a percentage of the optical density on the ipsilateral side compared with the contralateral control side.

### Western blot analysis

Brain tissues were prepared for western blotting as previously described [14, 20]. Briefly, rat SN tissues were homogenized and centrifuged at 4°C for 20 min at 14,000 g. The supernatant was transferred to a fresh tube and the concentration was determined using a BCA kit (Bio-Rad Laboratories, Hercules, CA). Proteins were separated using gel electrophoresis and transferred on to polyvinylidene difluoride membranes (Millipore) using an electrophoretic transfer system (Bio-Rad Laboratories). Membranes were incubated overnight at 4°C with specific primary antibodies: anti-β-actin (1:4000; Cell Signaling, Beverly, MA), anti-CNTF (1:1000; R&D systems) and anti-CNTFRα (1:10000; R&D systems). After washing, membranes were incubated with secondary antibodies (Amersham Biosciences, Piscataway, NJ) and the blots were developed with ECL western blotting detection reagents (Amersham Biosciences). For semi-quantitative analyses, the density of the immunoblot bands was measured with the Computer Imaging Device and accompanying software (Fuji Film, Tokyo, Japan).

### Statistical analysis

All values are expressed as mean ± standard error of the mean (SEM). Multiple comparisons among the groups were performed by one-way analysis of variance (ANOVA) followed by Tukey *post-hoc* analysis. All statistical analyses were performed using the Sigma Stat software (Systat Software, San Leandro, CA).

## Results

### hRheb(S16H) transduction in the substantia nigra pars compacta (SNpc) of normal rat brains

**Fig 1:**
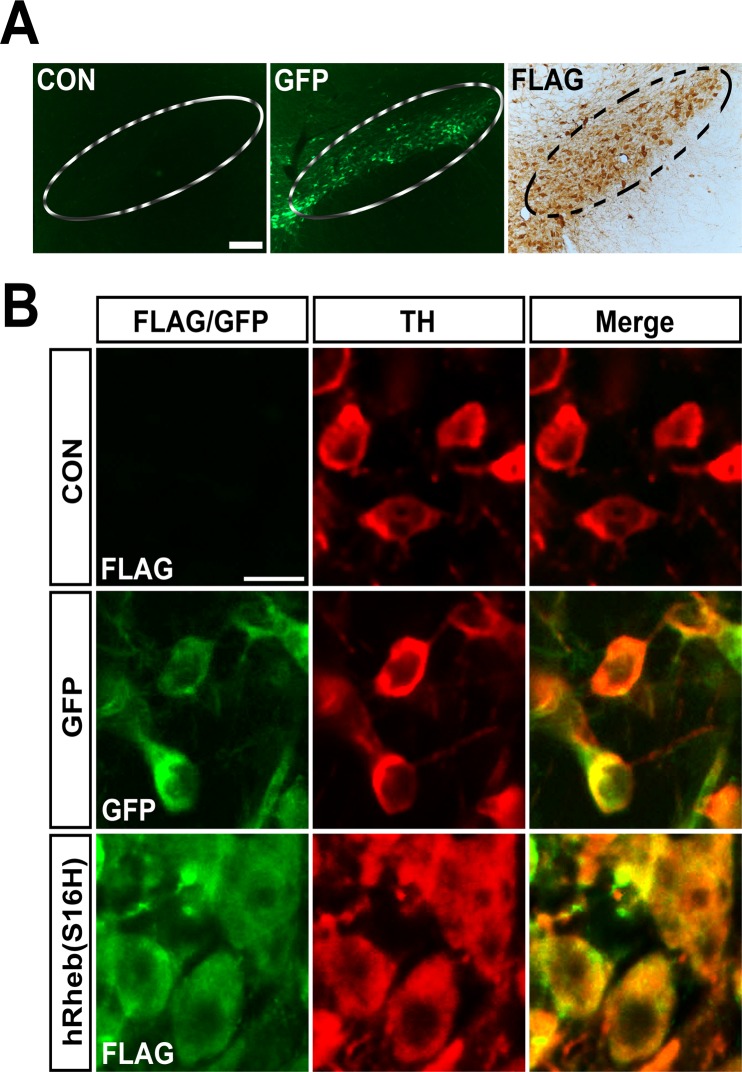
hRheb(S16H) transduction in SNpc of normal adult rats (A) Animals received a unilateral injection of AAV-GFP or AAV-hRheb(S16) in the SN and sacrificed 4 weeks later. AAV-hRheb(S16H)-treated sections were immunolabeled with FLAG. No expression was found in the non-injected control side (CON), but expression of GFP and FLAG by each viral vector injection is found in the ipsilateral side of SNpc of normal brains. Inside areas of the dotted lines indicate the SNpc. Scale bars, 200 μm. (B) Immunofluorescence double labeling for GFP (green) and TH (red), or FLAG (green) and TH shows that transgene expression is identifiable within dopaminergic neurons in the SN. Scale bar, 20 μm (n = 4, each group).

The rat brains were removed 4 weeks after the intranigral injection of AAV-GFP or AAV-hRheb(S16H). Brain sections were processed to determine the transduction of SNpc dopaminergic neurons by viral injection (Fig. 1). Consistent with previous results [12, 14], hRheb(S16H) and GFP transduction of SNpc dopaminergic neurons were confirmed by immunolabeling for FLAG and immediate observation of GFP expression, respectively (Fig. 1A). Transduction of dopaminergic neurons by AAV-GFP and AAV-hRheb(S16H) was confirmed by double-immunolabeling with GFP and TH, or FLAG and TH, respectively (Fig. 1B). hRheb(S16H)-transduced dopaminergic neurons showed the morphological changes compared with GFP-positive neurons, suggesting that hRheb(S16H) expression induced neurotrophic effects in dopaminergic neurons, as previously described [12, 14].

### Increases in the levels of CNTF and CNTFRα by hRheb(S16H) transduction of dopaminergic neurons *in vivo*

**Fig 2:**
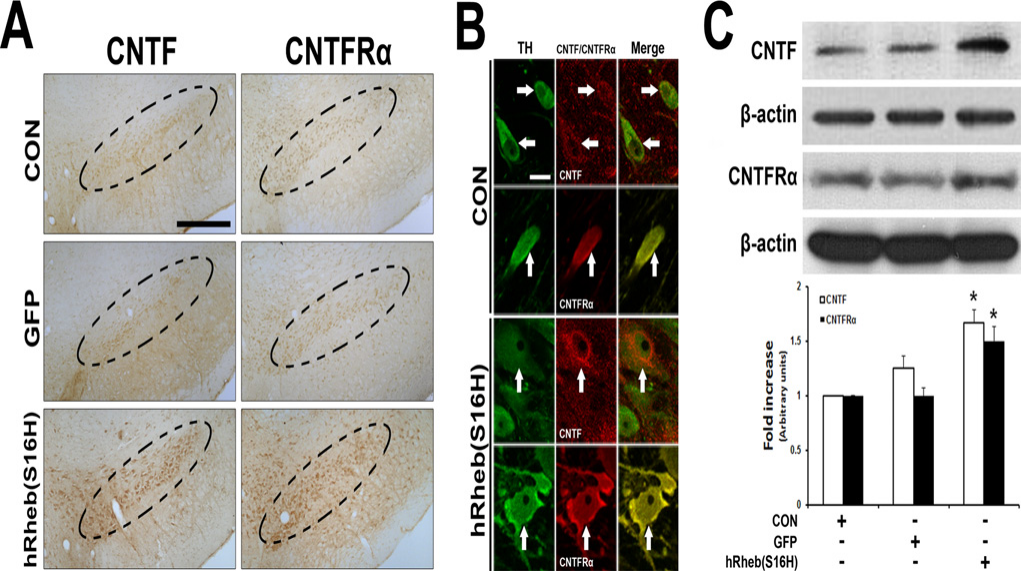
Induction of CNTF and CNTFRα by hRheb(S16H) transduction of dopaminergic neurons in the SN of rat brains. (A) The increase in CNTF and CNTFRα expression by hRheb(S16H) transduction of dopaminergic neurons in the SN of rat brains. Inside areas of the dotted lines indicate the SNpc. CON indicates the non-injected control side. Scale bars, 250 μm. (B) The increases in CNTF and CNTFRα expression in dopaminergic neurons by hRheb(S16H) transduction. White arrows indicate dopaminergic neurons (TH, green) merged with CNTF or CNTFRα (red) in the SN. Note that hRheb(S16H) transduction of dopaminergic neurons apparently increased the levels of CNTF and CNTFRα expression in dopaminergic neurons. Scale bar, 20 μm. (C) Consistent with the immunostaining results, western blotting showed increased levels of CNTF and CNTFRα in the SN 4 weeks after AAV-hRheb(S16) treatment, compared with controls. The histogram results show a quantitative analysis of density of the CNTF and CNTFRα bands normalized to β-actin for each sample. All values are expressed as mean ± SEM (*p < 0.01, significantly different from CON; One-way ANOVA and Tukey *post-hoc* analysis; n = 4, each group).

To examine whether hRheb(S16H) expression induced an increase in the levels of CNTF and CNTFRα in the SN, brain sections were immunostained with anti-CNTF or anti-CNTFRα 4 weeks after the intranigral injection of AAV-GFP or AAV-hRheb(S16H). Immunoperoxidase staining showed that the increases in CNTF and CNTFRα were observed in the SNpc following hRheb(S16H) transduction of dopaminergic neurons, compared with the contralateral control side and GFP-treated rats (Fig. 2A). The increased CNTF and CNTFRα were localized within dopaminergic neurons in the SN (Fig. 2B). Consistent with the immunostaining results, western blot analysis showed that hRheb(S16H) transduction of dopaminergic neurons significantly increased both CNTF and CNTFRα expression in the SN, compared with the control groups (Fig. 2C; *p* < 0.01).

### hRheb(S16H)-induced CNTF and CNTFRα contribute to the protection of the nigrostriatal dopaminergic projection in a neurotoxin model of PD

**Fig 3:**
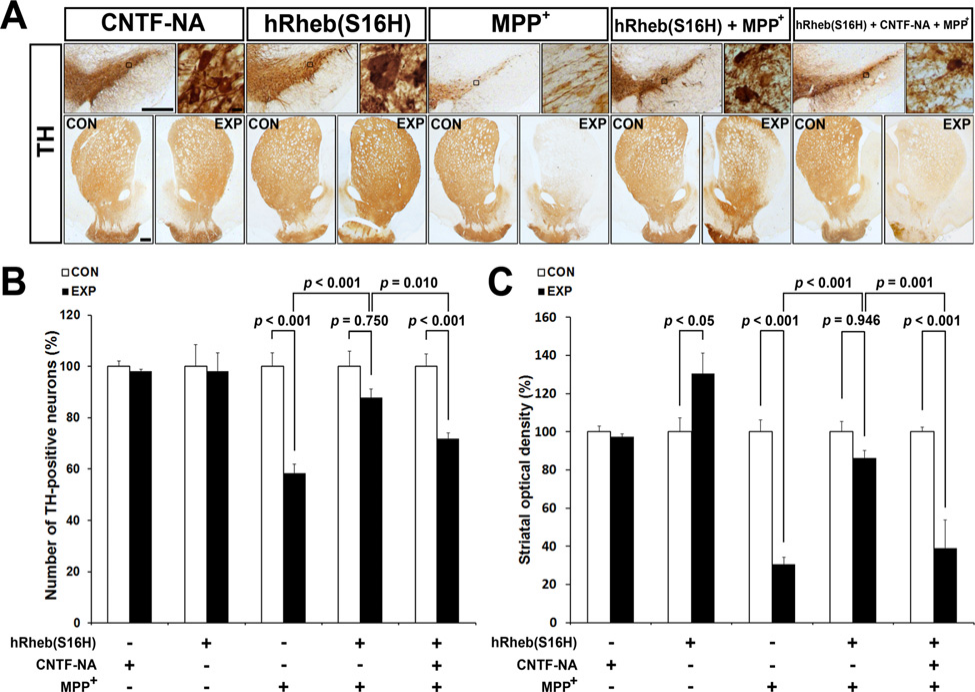
An increase in CNTF contributes to the hRheb(S16H)-induced neuroprotection of the nigrostriatal dopaminergic projection. (A) Rats received an intranigral injection of neutralizing antibodies for CNTF 3 weeks after AAV-hRheb(S16H) injection, and then they received an intra-MFB injection of MPP^+^ 10 min after treatment with neutralizing antibodies. Representative coronal sections of TH immunoperoxidase staining showed that hRheb(S16H) transduction of dopaminergic neurons induced neurotrophic effects as previously described [12, 14], protected the nigrostriatal dopaminergic projection from MPP^+^-induced neurotoxicity. However, treatment with neutralizing antibodies for CNTF (CNTF-NA) attenuated the neuroprotective effects of hRheb(S16H)-induced neuroprotection. Neutralizing antibodies alone were not neurotoxic in normal brains. CON, contralateral control side; EXP, ipsilateral injected side. Scale bars, 500 μm and 10 μm in the SN, respectively, and 500 μm in the STR. (B and C) The quantitative analysis of TH-positive neurons and fibers. Consistent with the immunostaining results, CNTF neutralization significantly inhibited hRheb(S16H)-induced neuroprotection in the MPP^+^-treated model of PD. All values are expressed as a percentage of the results in the ipsilateral SN and STR, respectively, compared with the contralateral controls (One-way ANOVA and Tukey *post-hoc* analysis; n = 4, each group).

We next examined whether hRheb(S16H)-induced CNTF and CNTFRα were neuroprotective in a neurotoxin model of PD. Rats received a unilateral injection of neutralizing antibodies against CNTF into the SN 3 weeks after AAV-hRheb(S16H) treatment and then received an injection of MPP^+^ into the MFB. Brain tissues treated with either neutralizing antibodies or MPP^+^ alone were used as a control for neurotoxicity. Immunostaining for TH, which was confirmed 1 week after the intranigral injection, showed that rats treated with CNTF neutralizing antibodies alone had no change in the number of TH-positive neurons or optical density of striatal TH immunostaining (Fig. 3A). However, the protective effects of hRheb(S16H) against MPP^+^-induced neurotoxicity were inhibited by its treatment (Fig. 3A). When quantified and expressed as a percentage of TH-positive neurons in the ipsilateral SN compared to the contralateral control, there were 58% and 88% TH-positive neurons in rats injected with MPP^+^ alone and MPP^+^ in the presence of hRheb(S16H), respectively (Fig. 3B; *p* < 0.001 and *p* = 0.750, respectively, *vs*. contralateral controls). This indicated that hRheb(S16H) was neuroprotective in the MPP^+^-treated animal model of PD (Fig. 3B; *p* < 0.001 *vs*. MPP^+^ alone). The administration of neutralizing antibodies for CNTF significantly attenuated the hRheb(S16H)-induced neuroprotection in the MPP^+^-treated model of PD [Fig. 3B; *p* < 0.001 *vs*. contralateral controls, *p* = 0.010 *vs*. hRheb(S16H) + MPP^+^]. As previously described [12, 14], hRheb(S16H) transduction of dopaminergic neurons significantly increased TH immunoreactivity in the STR, compared with contralateral controls (Figs. 3A and C; *p* < 0.05 *vs*. contralateral controls). Similar to the results in the SN, there were 30% and 86% TH-positive fibers in rats injected with MPP^+^ alone and MPP^+^ in the presence of hRheb(S16H), respectively (Fig. 3C; *p* < 0.001 and *p* = 0.946, respectively, *vs*. contralateral controls). This further indicated hRheb(S16H)-induced neuroprotection in the MPP^+^-treated model of PD (Fig. 3C; *p* < 0.001 *vs*. MPP^+^ alone). However, CNTF neutralization significantly inhibited hRheb(S16H)-induced neuroprotection [Fig. 3C; *p* < 0.001 *vs*. contralateral controls, *p* = 0.001 *vs*. hRheb(S16H) + MPP^+^].

**Fig 4:**
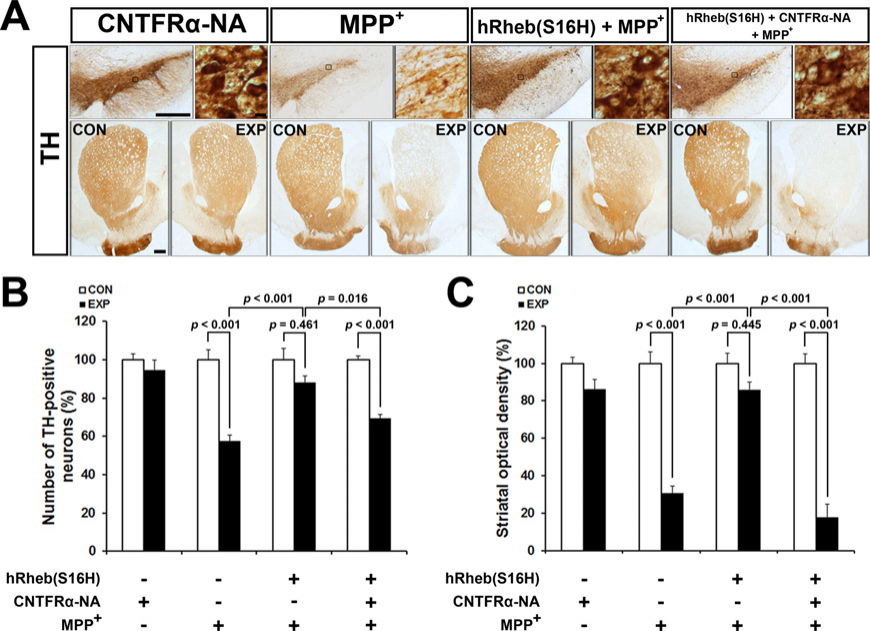
CNTFRα neutralization inhibits the protective effects of the nigrostriatal dopaminergic projection by hRheb(S16H) transduction against MPP^+^-induced neurotoxicity. (A) Neutralizing antibodies for CNTFRα (CNTFRα-NA) were injected in rats 3 weeks after the intranigral injection of AAV-hRheb(S16H). Following this, rats were injected into the MFB with MPP^+^. Representative coronal sections of the SN and STR of TH immunoperoxidase staining showed that CNTFRα neutralization attenuated the protective effects induced by hRheb(S16H) against MPP^+^ neurotoxicity. CON, contralateral control side; EXP, ipsilateral injected side. Scale bars represent 500 μm and 10 μm in the SN, respectively, and 500 μm in the STR. (B and C) The quantitative analysis of TH-positive neurons and fibers demonstrated that treatment with neutralizing antibodies for CNTFRα significantly decreased the hRheb(S16H)-induced neuroprotection on the nigrostriatal projection. All values are expressed as a percentage of the results in the ipsilateral SN and STR, respectively, compared with the contralateral controls (One-way ANOVA and Tukey *post-hoc* analysis; n = 4, each group).

To assess the role of CNTFRα in hRheb(S16H)-induced neuroprotection, we further analyzed additional rats that received an intranigral injection of neutralizing antibodies against CNTFRα (Fig. 4A). Similar to CNTF neutralization, this treatment significantly attenuated hRheb(S16H)-induced neuroprotection in both dopaminergic neurons and fibers in the MPP^+^-treated model of PD [Figs. 4B and C; *p* = 0.016 and *p* < 0.001 for TH-positive neurons and fibers, respectively, *vs*. hRheb(S16H) + MPP^+^]. However, anti-CNTFRα alone did not significant effect neurotoxicity (Figs. 4B and C; *p* = 0.946 and *p* = 0.299 for TH-positive neurons and fibers, respectively, *vs*. contralateral controls). Therefore, these results indicate that the activation of CNTF/CNTFRα signaling pathway by hRheb(S16H) transduction of dopaminergic neurons may contribute to the neuroprotection on the nigrostriatal dopaminergic projection in the adult brain.

## Discussion

Rheb is a small GTPase belonging to the Ras superfamily of guanine nucleotide-binding proteins. It is a key upstream regulator of mTORC1 activity, which is involved in multiple cellular functions, including protein synthesis, cell growth, proliferation, survival, and synaptic plasticity [21–24]. Our previous studies have shown that a constitutively active form of Rheb, such as hRheb(S16H), could induce neurotrophic effects in SNpc dopaminergic neurons, stimulate the production of dopamine in the nigrostriatal dopaminergic system, and induce neuroprotective and restorative effects via the activation of the mTORC1 signaling pathway in animal models of PD [12, 13]. In addition, the present study shows an additional mechanism of hRheb(S16H)-induced neuroprotection in a neurotoxin model of PD, suggesting that hRheb(S16H) transduction of dopaminergic neurons may have therapeutic potential as a treatment for PD.

**Fig 5:**
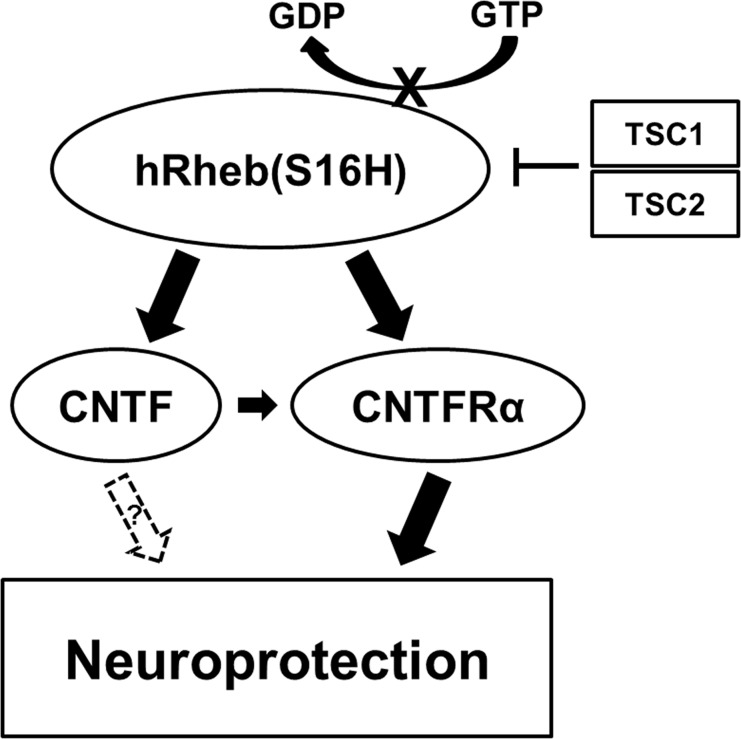
Schematic representation of hRheb(S16H)/CNTF and CNTFRα signaling pathways. The serine at position 16 of hRheb is sensitive to tuberous sclerosis complex (TSC) GTPase activation. hRheb(S16H), which has a mutation of the serine to histidine, is resistant to this activation and consequently cause its persistent GTP-bound, activated state [12]. The accumulation of hRheb(S16H) stimulates the expression of CNTF and its receptor in dopaminergic neurons, which can consequentially protect the nigrostriatal dopaminergic projection from neurotoxicity, even though it is obscure whether CNTF produced by hRheb(S16H) is involved in other potential mechanisms for protection of dopaminergic neurons (dotted arrow).

CNTF immunoreactivity is attenuated in dopaminergic neurons in the SN of PD patients [8]. Moreover, levels of CNTF and CNTFRα could increase in the brain following injury, such as focal cerebral ischemia and intracerebral hemorrhage [5–7]. These results suggest that the activation of survival signaling pathway by the induction of neurotrophic factors through an auto-/paracrine system after brain damage may be compensatory responses against neurodegeneration, which could be involved in PD [11], although it cannot fully protect the neurons in damaged brain regions. Furthermore, while neutralizing antibodies for CNTF and CNTFRα were not neurotoxic in this study, these data support the hypothesis that functional suppression of CNTF/CNTFRα signaling pathway may be involved in the neurodegeneration of dopaminergic neurons following neurotoxin treatment in the brain. Therefore, the enough supplies of neurotrophic factors such as CNTF could be crucial for the protection and maintenance of the nigrostriatal dopaminergic projection in the adult brain. However, similar to GDNF and BDNF [14], systemic delivery of CNTF as a therapeutic agent is blocked by the blood-brain barrier and intracerebral injection is not therapeutically valid due to its short half-life [25]. Nevertheless it has been shown to be neuroprotective against the degeneration of dopaminergic neurons [8, 10, 11]. These results suggest that the development of novel strategies for production or delivery of CNTF into the target region may be useful for prevention and therapy in PD. The results in the present study showed that viral vector-mediated hRheb(S16H) transduction increased the expression of CNTF and CNTFRα in dopaminergic neurons in the SN of rat brains (Fig. 2). Furthermore, treatment with specific neutralizing antibodies for CNTF and CNTFRα attenuated the protective effects of hRheb(S16H) against MPP^+^-induced neurotoxicity in the adult brain (Figs. 3 and 4). These results suggest that the activation of CNTF/CNTFRα signaling pathway contributes to hRheb(S16H)-induced neuroprotection; however, we cannot exclude the possibility that hRheb(S16H)-induced CNTF may be involved in other potential mechanisms, such as the induction of other neurotrophic factors, against neurotoxicity in dopaminergic neurons (Fig. 5).

In conclusion, in addition to the important role of hRheb(S16H) as an activator for the production of GDNF and BDNF in adult dopaminergic neurons [14], the present results show that specific gene delivery hRheb(S16H) can activate CNTF/CNTFRα signaling pathway in dopaminergic neurons in the SN and protect against MPP^+^-induced neurotoxicity. This may provide a useful therapeutic strategy for the neuroprotection of the nigrostriatal dopaminergic projection in the adult brain.
