# Correction: Activation of CNTF/CNTFRα Signaling Pathway by hRheb(S16H) Transduction of Dopaminergic Neurons *In Vivo*

**DOI:** 10.1371/journal.pone.0126450

**Published:** 2015-04-17

**Authors:** The PLOS ONE Staff

There are errors in the author affiliations. The affiliations should appear as shown here: Kyoung Hoon Jeong^1,2^, Jin Han Nam^4^, Byung Kwan Jin,^4,5^ and Sang Ryong Kim^1,2,3,6^

1 School of Life Sciences, Kyungpook National University, Daegu, Korea. 2 BK21 plus KNU Creative BioResearch Group, Kyungpook National University, Daegu, Korea. 3 Institute of Life Science & Biotechnology, Kyungpook National University, Daegu, Korea. 4 Neurodegeneration Control Research Center, Kyung Hee University, Seoul, Korea. 5 Department of Biochemistry & Molecular Biology, School of Medicine, Kyung Hee University, Seoul, Korea. 6 Brain Science and Engineering Institute, Kyungpook National University, Daegu, Korea.

There is an error in Fig 2, “Induction of CNTF and CNTFRα by hRheb(S16H) transduction of dopaminergic neurons in the SN of rat brains.” Please see the corrected Fig 2 and its legend here:

**Fig 2:**
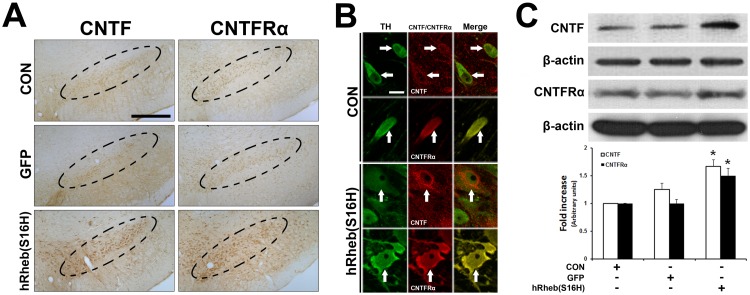
Induction of CNTF and CNTFRα by hRheb (S16H) transduction of dopaminergic neurons in the SN of rat brains. (A) The increase in CNTF and CNTFRα expression by hRheb(S16H) transduction of dopaminergic neurons in the SN of rat brains. Inside areas of the dotted lines indicate the SNpc. CON indicates the non-injected control side. Scale bars, 250 μm. (B) The increases in CNTF and CNTFRα expression in dopaminergic neurons by hRheb(S16H) transduction. White arrows indicate dopaminergic neurons (TH, green) merged with CNTF or CNTFRα (red) in the SN. Note that hRheb(S16H) transduction of dopaminergic neurons apparently increased the levels of CNTF and CNTFRα expression in dopaminergic neurons. Scale bar, 20 μm. (C) Consistent with the immunostaining results, western blotting showed increased levels of CNTF and CNTFRα in the SN 4 weeks after AAV-hRheb(S16) treatment, compared with controls. The histogram results show a quantitative analysis of density of the CNTF and CNTFRα bands normalized to β-actin for each sample. All values are expressed as mean ± SEM (*p <0.01, significantly different from CON; One-way ANOVA and Tukey *post-hoc* analysis; n = 4, each group)
